# Trends and projections of polycystic ovary syndrome burden in China: Insights from the Global Burden of Disease Study 2021

**DOI:** 10.1371/journal.pone.0332082

**Published:** 2025-09-18

**Authors:** Huanghui Qin, Hang Liu, Junming Sun, Liang Cai, Yi Tan, Mingze Li, Yubo Xiao, Lanyu Li

**Affiliations:** 1 Guangxi Key laboratory of Diabetic Systems Medicine, Guilin Medical University, Guilin, Guangxi, China; 2 School of Medical Laboratory Science, Hunan University of Medicine, Huaihua, Hunan, China; 3 Laboratory Animal Center, Guangxi Medical University, Nanning, Guangxi, China; 4 Guidong People’s Hospital of Guangxi Zhuang Autonomous Region, Wuzhou, China; 5 Georgia Institute of Technology, North Avenue, Atlanta, Georgia United States of America; Lewis Katz School of Medicine at Temple University, UNITED STATES OF AMERICA

## Abstract

**Background:**

Polycystic ovary syndrome (PCOS) is a prevalent endocrine disorder among women of reproductive age, associated with reproductive, metabolic, and psychological complications. In China, the burden of PCOS remains poorly characterized, particularly amid changing demographics and lifestyle patterns. This study evaluates trends in PCOS incidence, prevalence, and disability-adjusted life-years (DALYs) from 1990 to 2021 and projects the future burden through 2035.

**Methods:**

Data on PCOS incidence, prevalence, and DALYs for Chinese women aged 10–54 years were extracted from the Global Burden of Disease Study 2021. Age-specific and age-standardized rates were calculated. Temporal trends were assessed using estimated annual percentage changes (EAPCs), and decomposition analysis quantified contributions of epidemiological changes, population growth, and aging. Projections through 2035 were based on current trends.

**Results:**

From 1990 to 2021, PCOS incidence and prevalence showed significant increases, especially in younger age groups. Among 10–14-year-olds, incidence rose from 73,615 cases (95% UI: 35,399−124,529) to 128,219 cases (95% UI: 65,776–211,113), while prevalence increased from 124,220 (95% UI: 59,649–211,274) to 216,398 cases (95% UI: 110,028–357,026). Age-standardized rates are projected to rise to 70.82 (95% CI: 45.39–96.26) and 1,661.80 (95% CI: 1,467.99–1,855.62) per 100,000 by 2035, respectively. Decomposition analysis showed epidemiological changes as the primary driver of increased burden.

**Conclusions:**

The burden of PCOS in China has risen substantially over three decades and is projected to escalate further. Marked increases in PCOS incidence and prevalence were observed among younger age groups, indicating an earlier onset or diagnosis. These findings highlight a shifting burden toward younger age groups and underscore the importance of age-specific surveillance and prevention strategies to address the evolving epidemiology of PCOS in China.

## Introduction

Polycystic ovary syndrome (PCOS) is the most common endocrine disorder affecting women of reproductive age globally, with an estimated prevalence ranging between 6% and 19% depending on the diagnostic criteria used [1–3]. PCOS is characterized by reproductive dysfunction, including menstrual irregularities and infertility, alongside metabolic disturbances such as obesity, insulin resistance, and type 2 diabetes [4,5]. Beyond its reproductive and metabolic consequences, PCOS imposes a substantial psychological burden, with increased risks of depression, anxiety, and reduced quality of life [6]. PCOS often manifests during adolescence, with clinical features such as menstrual irregularities and hyperandrogenism typically emerging shortly after menarche. While symptoms may improve with age, associated metabolic complications—including insulin resistance, dyslipidemia, and increased cardiometabolic risk—often persist into adulthood and beyond [7]. Despite its far-reaching impacts, PCOS remains underdiagnosed and undertreated, particularly in low-resource settings [8].

In China, rapid socioeconomic development, urbanization, and lifestyle transitions over the past three decades have led to rising obesity rates and increasing physical inactivity, particularly among younger populations. These trends are significant drivers of insulin resistance and hyperandrogenism, key pathophysiological components of PCOS [9,10]. Moreover, improvements in healthcare access and diagnostic capabilities have likely contributed to higher detection rates of PCOS in recent years. However, data on its trends and burden in China remain limited. Understanding the age-specific dynamics of PCOS burden is critical to inform targeted public health strategies. Previous studies have investigated the global and regional burden of PCOS using GBD data, indicating increasing prevalence and associated metabolic risks over time. For example, Zhang et al. (2024) reported global and regional PCOS trends from 1990 to 2019, but did not provide projections or age-stratified insights. However, few studies have focused specifically on China or assessed future trends in disease burden through decomposition and forecasting analyses. Our study addresses this gap by offering a comprehensive, age-specific assessment of PCOS incidence, prevalence, and DALYs from 1990 to 2021, and providing projections to 2035 [3,11].

This study aims to provide a comprehensive assessment of the trends and future projections of PCOS burden among Chinese women aged 10–54 years from 1990 to 2035. Using data from the Global Burden of Disease (GBD) Study 2021, we analyzed trends in incidence, prevalence, and disability-adjusted life-years (DALYs) and quantified the contributions of epidemiological changes, population growth, and population aging. Additionally, we provide projections of PCOS burden through 2035 to anticipate the impact of evolving demographic and lifestyle trends.

## Methods

### Study population and data collection

This study utilized data from the GBD Study 2021, a comprehensive, systematic analysis providing age-, sex-, and location-specific estimates of diseases and injuries globally [12]. Data on the incidence, prevalence, and DALYs for PCOS among Chinese women aged 10–54 years from 1990 to 2021 were extracted [13]. The GBD framework applies standardized methodologies to synthesize data from multiple sources, including health surveys, hospital records, claims databases, and published studies. Estimates were adjusted to ensure comparability across time and locations using the DisMod-MR 2.1 Bayesian meta-regression tool developed by the Institute for Health Metrics and Evaluation (IHME) [14]. This model accounts for variations in data sources, diagnostic definitions, and sampling designs, and applies covariate smoothing, internal consistency checks, and statistical corrections to harmonize estimates across years and geographic regions. GBD adjusts for differences in diagnostic criteria through statistical models, such as DisMod-MR 2.1, which uses data cross walking and covariate adjustment to harmonize estimates across studies with differing definitions (e.g., NIH, Rotterdam, AE-PCOS) [15].

Age groups were categorized into 10–14 years, 15–19 years, 20–24 years, 25–29 years, 30–34 years, 35–39 years, 40–44 years, 45–49 years, and 50–54 years to examine age-specific trends. Population data were derived from the GBD Study, which uses United Nations Population Division estimates as a reference [16].

### Ethics statement

This study utilized publicly available, de-identified, aggregate data from the Global Burden of Disease Study 2021 (GBD 2021). As no individual-level patient data were accessed, generated, or analyzed, and the data used are fully anonymized and publicly accessible for research purposes, this study did not require institutional review board (IRB) approval or informed consent. The need for ethics review was waived by the nature of the data source.

### Statistical analysis

Trends in the burden of PCOS were assessed through age-standardized and age-standardized incidence rates (ASIR), age-standardized prevalence rates (ASPR), and age-standardized DALYs rates(ASDR). Temporal changes were analyzed using estimated annual percentage change (EAPC), a widely accepted metric for quantifying trends over time [11]. EAPC was calculated using a generalized linear regression model, where the natural logarithm of the rate was regressed against the calendar year:


In(rate)=α+β×year+ϵ

The EAPC and its 95% confidence interval (CI) were derived as:


$$\text{EAPC}=\text{100}\times (e^{\beta }-1)$$

A positive EAPC indicates an increasing trend, while a negative EAPC reflects a decline. Statistical significance was determined based on the 95% CI of the EAPC not crossing zero [17].

To identify the underlying drivers of changes in PCOS burden, decomposition analysis was performed to quantify the contributions of epidemiological changes, population growth, and population aging to variations in incidence, prevalence, and DALYs. The DALYs were calculated by combining the years lived with disability (YLDs) for PCOS with the years of life lost (YLLs) due to this condition, though YLLs are typically minimal for PCOS as it is rarely directly fatal [11,18]. This approach allows differentiation between changes attributable to population structure (size and age) and changes in disease risk or healthcare practices over time.

Decomposition analysis was used to attribute changes in PCOS burden to three contributing factors: population growth, population aging, and epidemiological shifts. The method is based on a stepwise counterfactual approach that sequentially isolates the impact of each component [19]. Specifically, total change in burden (△Y) is expressed as:


△Y=△P+△A+△E

where △P represents the change attributable to population growth, estimated by comparing the observed burden to a scenario where population size is held constant. △A represents the effect of population aging, calculated by allowing age distribution to change while holding age-specific rates constant. △E captures the contribution of epidemiological changes, defined as changes in age-specific rates with the population structure held constant.

To assess the temporal dynamics of PCOS burden and generate projections to 2035, we employed an Age–Period–Cohort (APC) analysis, which decomposes disease trends into three components:

Age Effect (α): Reflects age-specific differences in disease risk, capturing the impact of biological and demographic factors.

Period Effect (β): Represents temporal changes that affect all age groups simultaneously, accounting for shifts in healthcare, public awareness, and diagnostic practices.

Cohort Effect (γ): Captures generational differences, indicating how exposure to risk factors varies among birth cohorts.

The APC model is mathematically expressed as:


$$Y_{ijk}=\mu +\alpha_{i}+\beta_{j}+\gamma_{k}+\epsilon_{ijk}$$

where Y_*ijk*_ represents the observed disease burden for age group i, period j, and cohort k; is the overall intercept; and ∊_*ijk*_ is the random error term [20]. The BAPC model was used for projection, leveraging Markov Chain Monte Carlo (MCMC) simulations. Convergence of MCMC chains was evaluated using the Gelman-Rubin diagnostic [21]. These methods ensured a robust assessment of temporal trends, stratified by age, period, and cohort effects.

Future projections of PCOS incidence, prevalence, and DALYs through 2035 were generated using time-series forecasting methods based on current trends. The forecasts assumed a continuation of existing demographic and epidemiological patterns, with 95% uncertainty intervals (UIs) derived through simulation to account for model variability [22].

All analyses adhered to the GBD Study’s standardized statistical protocols, ensuring methodological consistency and comparability. Data processing and visualization were conducted using R (version 4.1.2).

## Results

### Trends in incidence, prevalence and DALYs numbers of PCOS in China by age group

From 1990 to 2021, incidence numbers exhibited distinct age-specific trends (Table 1). In the 10–14 years group, numbers increased substantially from 73,615 (95% UI: 35,399–124,529) to 128,219 (95% UI: 65,776–211,113). The 15–19 years group saw a modest rise from 107,312 (95% UI: 65,775–173,125) to 109,707 (95% UI: 67,434–175,302). Conversely, the 20–24 years group demonstrated a notable decline, with incidence numbers decreasing from 13,700 (95% UI: 5,704–33,400) in 1990–6,962 (95% UI: 2,807–17,824) in 2021. A similar reduction was observed in the 25–29 years group, where incidence fell from 6,619 (95% UI: 3,402–12,018) to 4,813 (95% UI: 2,430–9,120). In the 50–54 years age group, although incidence numbers remained low, a slight increase was recorded, rising from 117 (95% UI: 13–304) in 1990–307 (95% UI: 33–796) in 2021.

**Table 1:** The number of incidence cases and the ASIR of Polycystic Ovary Syndrome in 1990 and 2021, and its temporal trends from 1990 to 2021.

Age	1990		2021		1990–2021
	Number of cases (95%UI)	ASIR/100000(95% UI)	Number of cases (95%UI)	ASIR/100000(95% UI)	EAPC (95%CI)
10-14 years	73615 (35399, 124529)	148.74 (71.52, 251.61)	128219 (65776, 211113)	318.95 (163.62, 525.15)	2.30 (2.13, 2.47)
15-19 years	107312 (65775, 173125)	174.11 (106.72, 280.89)	109707 (67434, 175302)	317.17 (194.96, 506.81)	1.76 (1.55, 1.98)
20-24 years	13700 (5704, 33400)	21.25 (8.84, 51.79)	6962 (2807, 17824)	20.29 (8.18, 51.95)	−0.19 (−0.28, −0.10)
25-29 years	6619 (3402, 12018)	12.38 (6.36, 22.48)	4813 (2430, 9120)	11.78 (5.95, 22.32)	−0.09 (−0.16, −0.01)
30-34 years	2981 (1390, 4737)	7.06 (3.29, 11.22)	3921 (1798, 6240)	6.71 (3.08, 10.67)	−0.05 (−0.11, 0.01)
35-39 years	2346 (1120, 3820)	5.32 (2.54, 8.66)	2619 (1204, 4292)	5.08 (2.33, 8.32)	−0.05 (−0.09, 0.00)
40-44 years	1096 (482, 1882)	3.43 (1.51, 5.90)	1473 (617, 2570)	3.30 (1.38, 5.76)	−0.01 (−0.07, 0.04)
45-49 years	421 (84, 874)	1.72 (0.34, 3.59)	908 (177, 1859)	1.67 (0.33, 3.43)	0.04 (0.00, 0.07)
50-54 years	117 (13, 304)	0.52 (0.06, 1.35)	307 (33, 796)	0.51 (0.05, 1.33)	0.14 (0.08, 0.21)

Abbreviations: ASIR, age-standardized incidence rate; EAPC, estimate annual percentage change.

The prevalence of PCOS in China increased significantly across most age groups during the study period (Table 1). In the 10–14 years age group, prevalence numbers rose markedly from 124,220 (95% UI: 59,649–211,274) in 1990–216,398 (95% UI: 110,028–357,026) in 2021. For individuals aged 15–19 years, prevalence increased from 740,234 (95% UI: 488,240–1,082,036) to 814,741 (95% UI: 540,161–1,184,351). The 20–24 years group exhibited a similar upward trend, with prevalence numbers increasing from 1,041,926 (95% UI: 719,966–1,495,805) to 1,081,417 (95% UI: 745,235–1,535,505). In the 25–29 years group, prevalence grew from 916,080 (95% UI: 635,153–1,291,175) to 1,301,756 (95% UI: 905,869–1,833,593). Despite these increases, older age groups had lower prevalence numbers. For the 50–54 years group, prevalence rose steadily from 83,652 (95% UI: 56,349–123,465) in 1990–379,602 (95% UI: 253,770–548,641) in 2021.

The DALYs attributable to PCOS in China also showed significant growth, particularly in younger age groups (Table 3). Among individuals aged 10–14 years, DALYs numbers increased from 1,079 (95% UI: 405–2,451) in 1990–1,887 (95% UI: 742–4,178) in 2021. For the 15–19 years group, DALYs numbers rose from 6,633 (95% UI: 2,855–14,263) to 7,305 (95% UI: 3,194–15,860). In the 20–24 years group, DALYs increased from 9,226 (95% UI: 3,910–19,122) to 9,717 (95% UI: 4,296–20,202). A similar trend was observed in the 25–29 years group, where DALYs numbers grew from 7,875 (95% UI: 3,363–16,207) to 11,340 (95% UI: 4,861–23,780). In the 50–54 years group, although DALYs numbers remained relatively low, they increased notably from 712 (95% UI: 312–1,602) in 1990–3,219 (95% UI: 1,375–6,964) in 2021.

**Table 3:** The number of DALYs cases and the age-standardized DALYs rate of Polycystic Ovary Syndrome in 1990 and 2021, and its temporal trends from 1990 to 2021.

Age	1990		2021		1990–2021
	Number of cases (95%UI)	ASDR/100000 (95%UI)	Number of cases (95%UI)	ASDR/100000 (95%UI)	EAPC (95%CI)
10-14 years	1079 (405, 2451)	2.18 (0.82, 4.95)	1887 (742, 4178)	4.69 (1.85, 10.39)	2.31 (2.14, 2.48)
15-19 years	6633 (2855, 14263)	10.76 (4.63, 23.14)	7305 (3194, 15860)	21.12 (9.23, 45.85)	1.99 (1.75, 2.23)
20-24 years	9226 (3910, 19122)	14.31 (6.06, 29.65)	9717 (4296, 20202)	28.32 (12.52, 58.89)	2.22 (1.95, 2.49)
25-29 years	7875 (3363, 16207)	14.73 (6.29, 30.31)	11340 (4861, 23780)	27.75 (11.90, 58.19)	2.27 (2.06, 2.49)
30-34 years	6357 (2743, 13583)	15.06 (6.50, 32.17)	15750 (6775, 33540)	26.94 (11.59, 57.36)	2.13 (2.01, 2.26)
35-39 years	6408 (2765, 13677)	14.53 (6.27, 31.00)	13596 (5836, 28377)	26.35 (11.31, 55.00)	2.01 (1.89, 2.13)
40-44 years	4543 (1950, 9563)	14.24 (6.11, 29.97)	11463 (4965, 23766)	25.70 (11.13, 53.28)	1.98 (1.82, 2.15)
45-49 years	3036 (1367, 6368)	12.45 (5.61, 26.12)	12166 (5424, 25032)	22.42 (10.00, 46.13)	2.01 (1.81, 2.22)
50-54 years	712 (312, 1602)	3.17 (1.39, 7.14)	3219 (1375, 6964)	5.39 (2.30, 11.66)	2.01 (1.84, 2.19)

Abbreviations: ASDR, age-standardized DALYs rates; EAPC, estimate the annual percentage change.

### Trends in incidence, prevalence and DALYs rate of PCOS in China by age group

**Fig 1:**
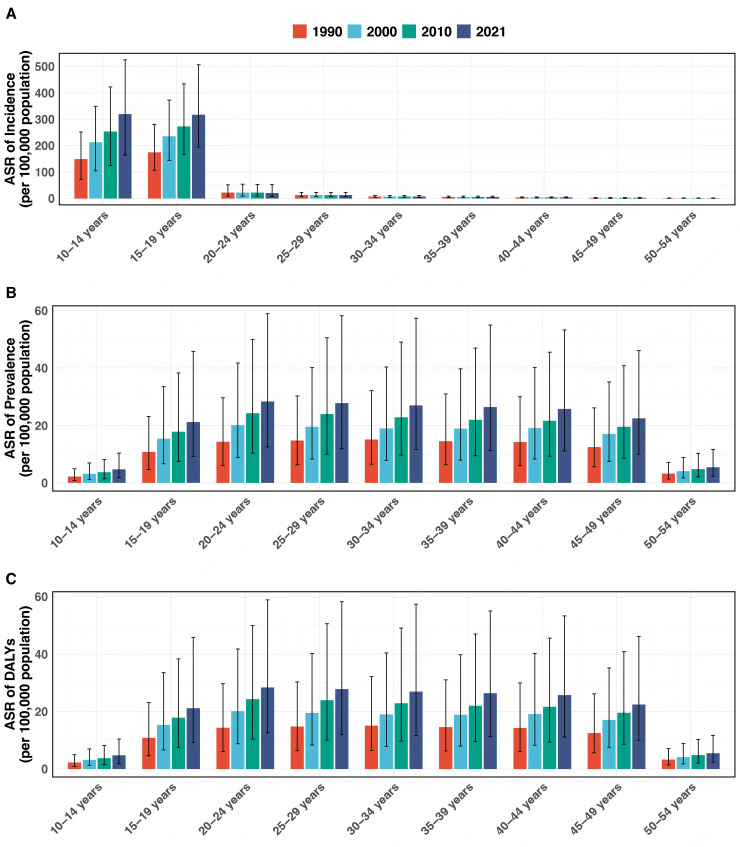
Trends in Incidence, Prevalence and DALYs rate of Polycystic Ovary Syndrome in China Aged 10–54 Years (1990–2021). Panel A shows the age-specific incidence rate (per 100,000 women), Panel B illustrates the age-specific prevalence rate (per 100,000 women), and Panel C displays the age-specific DALYs (per 100,000 women). The bars represent data from four distinct years: red for 1990, blue for 2000, green for 2010, and purple for 2021. Error bars indicate the 95% confidence intervals. Abbreviations: DALYs, disability-adjusted life years.

The ASIR of PCOS exhibited significant age-specific variations from 1990 to 2021 (Table 1, Fig 1A). In the 10–14 years group, ASIR rose from 148.74 (95% UI: 71.52–251.61) per 100,000 in 1990 to 318.95 (95% UI: 163.62–525.15) per 100,000 in 2021, with an estimated EAPC of 2.30 (95% CI: 2.13–2.47). Similarly, in the 15–19 years group, the ASIR increased from 174.11 (95% UI: 106.72–280.89) per 100,000 to 317.17 (95% UI: 194.96–506.81) per 100,000,EAPC 1.76(95% CI: 1.55–1.98). Conversely, slight declines were noted among older adolescents and young adults. In the 20–24 years group, the ASIR decreased marginally from 21.25 per 100,000 (95% UI: 8.84–51.79) to 20.29 per 100,000(95% UI: 8.18–51.95),EAPC −0.19(95% CI: −0.28 – −0.10), and among the 25–29 years group, the rate fell slightly from 12.38 per 100,000 (95% UI:6.36–22.48) to 11.78 per 100,000 (95% UI: 5.95–22.32),EAPC −0.09(95% CI: −0.16 – −0.01). For the 50–54 years group, ASIR remained low and showed a slight decrease from 0.52 per 100,000 (95%UI: 0.06–1.35) in 1990 to 0.51 per 100,000 (95% UI: 0.05–1.33) in 2021, with an EAPC of 0.14 (95% CI: 0.08–0.21).

The ASPR of PCOS also showed a consistent upward trend across all groups, with the largest increases recorded in younger individuals (Table 2, Fig 1B). For the 10–14 years age group, the ASPR rose from 250.98 per 100,000 (95% UI: 120.52–426.88) in 1990 to 538.30 per 100,000 (95% UI: 273.70–888.11) in 2021, with an EAPC of 2.30 (95% CI: 2.13–2.47). In the 15–19 years group, ASPR increased from 1201.02 per 100,000 (95% UI: 792.16–1755.59) to 2355.47 per 100,000 (95% UI: 1561.64–3424.03),EAPC 2.00(95% CI: 1.77–2.24). Among the 20–24 years group, the ASPR grew from 1,615.72 per 100,000 (95% CI: 1,116.45–2,319.55) to 3,152.21 per 100,000 (95% CI: 2,172.27–4,475.82),EAPC 2.20(95% CI: 1.93–2.46). For the 50–54 years group, ASPR remained comparatively low but increased steadily from 372.52 per 100,000 (95% UI: 250.94–549.83) in 1990 to 635.66 per 100,000 (95% UI: 424.95–918.72) in 2021 EAPC 2.02(95% CI: 1.85–2.20).

**Table 2:** The number of prevalence cases and the ASPR of Polycystic Ovary Syndrome in 1990 and 2021, and its temporal trends from 1990 to 2021.

Age	1990		2021		1990–2021
	Number of cases (95%UI)	ASPR/100000(95% UI)	Number of cases (95%UI)	ASPR/100000(95% UI)	EAPC (95%CI)
10-14 years	124220 (59649, 211274)	250.98 (120.52, 426.88)	216398 (110028, 357026)	538.30 (273.70, 888.11)	2.30 (2.13, 2.47)
15-19 years	740234 (488240, 1082036)	1201.02 (792.16, 1755.59)	814741 (540161, 1184351)	2355.47 (1561.64, 3424.03)	2.00 (1.77, 2.24)
20-24 years	1041926 (719966, 1495805)	1615.72 (1116.45, 2319.55)	1081417 (745235, 1535505)	3152.21 (2172.27, 4475.82)	2.20 (1.93, 2.46)
25-29 years	916080 (635153, 1291175)	1713.48 (1188.02, 2415.08)	1301756 (905869, 1833593)	3185.40 (2216.66, 4486.80)	2.25 (2.04, 2.46)
30-34 years	755818 (527187, 1063372)	1790.21 (1248.68, 2518.67)	1857399 (1301558, 2638253)	3176.70 (2226.05, 4512.19)	2.11 (1.99, 2.24)
35-39 years	767993 (541511, 1071157)	1740.97 (1227.56, 2428.22)	1618329 (1127261, 2313928)	3136.47 (2184.73, 4484.60)	2.00 (1.88, 2.12)
40-44 years	545151 (385075, 760960)	1708.17 (1206.59, 2384.39)	1368709 (958422, 1941039)	3068.28 (2148.52, 4351.29)	1.97 (1.81, 2.13)
45-49 years	360257 (253605, 505769)	1477.66 (1040.21, 2074.51)	1439168 (1013164, 2042213)	2652.35 (1867.24, 3763.75)	2.01 (1.80, 2.21)
50-54 years	83652 (56349, 123465)	372.52 (250.94, 549.83)	379602 (253770, 548641)	635.66 (424.95, 918.72)	2.02 (1.85, 2.20)

Abbreviations: ASPR, age-standardized prevalence rate; EAPC, estimate annual percentage change.

The DALYs due to PCOS in China also increased substantially over the study period, with the most pronounced growth observed in younger age groups (Table 3, Fig 1C). In the 10–14 years group, the DALYs rate rose from 2.18 per 100,000 (95% UI: 0.82–4.95) in 1990 to 4.69 per 100,000 (95% UI: 1.85–10.39) in 2021, with an EAPC of 2.31 (95% CI: 2.14–2.48). For the 15–19 years age group, the DALYs rate increased from 10.76 per 100,000 (95% UI: 4.63–23.14) to 21.12 per 100,000 (95% UI: 9.23–45.85),EAPC 1.99 (95% CI: 1.75–2.23). In the 20–24 years group, the DALYs rate grew markedly from 14.31 per 100,000 (95% CI: 6.06–29.65) to 28.32 per 100,000 (95% CI: 12.52–58.89),EAPC 2.22(95% CI: 1.95–2.49). For the 50–54 years group, the DALYs rate remained lower but demonstrated a modest increase from 3.17 per 100,000 (95% UI: 1.39–7.14) to 5.39 per 100,000 (95% UI: 2.30–11.66),EAPC 2.01(95% CI: 1.84–2.19).

### Decomposition analysis of factors influencing PCOS in China

**Fig 2:**
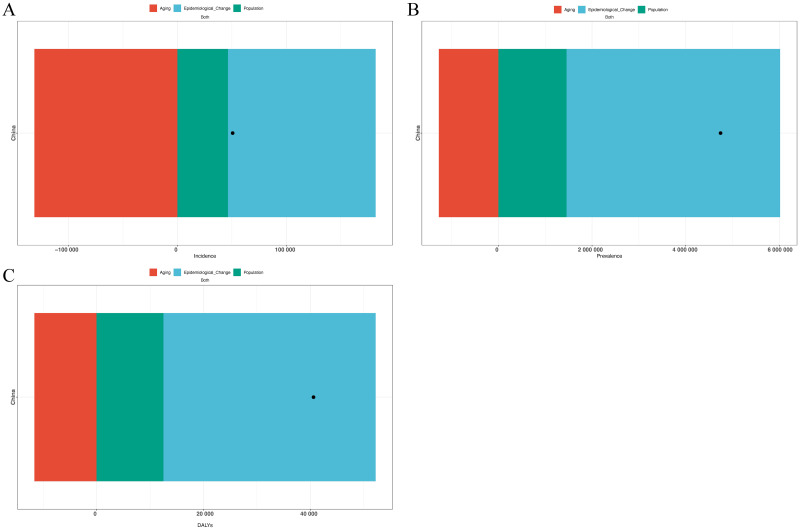
Decomposition of Changes in Polycystic Ovary Syndrome Burden Among Chinese Women. The changes are broken down into three contributing factors: aging (red), epidemiological change (blue), and population growth (green). Panel A shows the decomposition of changes in incidence, Panel B displays the decomposition of changes in prevalence, and Panel C illustrates the decomposition of changes in DALYs. The stacked bars represent the relative contribution of each factor, while the black dot indicates the net change resulting from the combined effects. Abbreviations: DALYs, disability-adjusted life years.

The decomposition analysis of factors contributing to the burden of PCOS in China revealed distinct trends across incidence, prevalence, and DALYs (Fig 2). For incidence, the overall increase was 50,724.15 cases. This change was predominantly driven by epidemiological changes, which contributed a significant rise of 135,613.72 cases (267.36%). Population growth added 46,417.64 cases (91.51%) (Fig 2A). In contrast, population aging had a negative contribution, reducing incidence by –131,307.21 cases (–258.87%).

For prevalence, the overall burden increased substantially by 4,742,190 cases. The primary driver was epidemiological changes, accounting for 4,557,401 cases (96.1%). Population growth contributed 1,454,881 cases (30.68%). Conversely, population aging reduced prevalence by –1,270,091 cases (–26.78%) (Fig 2B).

For DALYs, the total increase was 40,573.26 DALYs. The most significant contributor was epidemiological changes, which accounted for 39,682.84 DALYs (97.81%). Population growth further contributed 12,508.86 DALYs (30.83%). However, population aging resulted in a reduction of –11,618.45 DALYs (–28.64%) (Fig 2C).

### Trends and future projections of PCOS burden among Chinese women (1990–2035)

**Fig 3:**
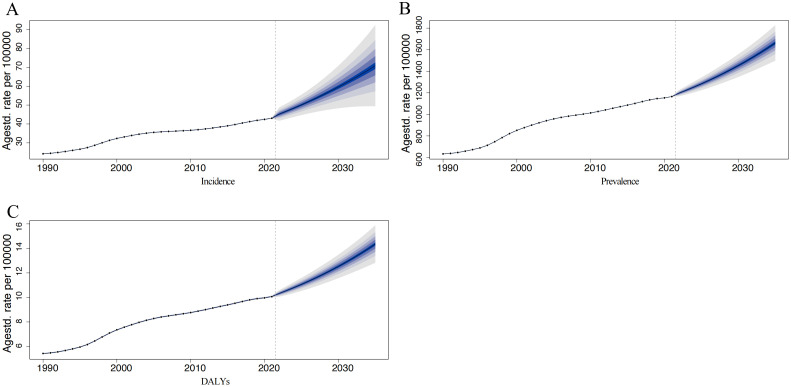
Trends and Future Projections of Polycystic Ovary Syndrome Burden Among Chinese Women (1990–2035). Panel A shows the age-standardized incidence rate (per 100,000), Panel B presents the age-standardized prevalence rate (per 100,000), and Panel C depicts the age-standardized DALYs rate (per 100,000). The solid black line represents observed rates, while the shaded areas indicate projections with 95% uncertainty intervals.

The predicted trends for PCOS in China from 2022 to 2035 reveal substantial increases in the burden of disease across incidence, prevalence, and DALYs (Fig 3). Projections indicate a marked acceleration post-2022, with the ASIR expected to rise from 45.22 (95% CI: 41.10–49.33) per 100,000 in 2022 to 70.82 (95% CI: 45.39–96.26) per 100,000 in 2035. Similarly, the ASPR of PCOS is projected to increase rapidly, rising to 1282.84 (95% CI: 1214.55–1351.12) per 100,000 by 2025 and further to 1,452.00 (95% CI: 1,328.57–1,575.44) per 100,000 by 2030. By 2035, ASPR is expected to reach 1,661.80 (95% CI: 1,467.99–1,855.62) per 100,000. The age-standardized DALYs rate, which was 10.07 (95% CI: 10.01–10.14) per 100,000 in 2021, is also projected to show a sustained upward trend, reaching 13.20 (95% CI: 11.84–14.57) per 100,000 by 2032 and 14.35 (95% CI: 12.54–16.15) per 100,000 by 2035.

## Discussion

Our study demonstrates substantial increases in PCOS incidence, prevalence, and DALYs among Chinese women aged 10–54 years (1990–2021), reflecting demographic shifts, lifestyle changes, and improved disease recognition. Projections indicate continued burden through 2035, underscoring the need for targeted public health strategies.

Younger groups (10–14 and 15–19 years) showed the most pronounced increases, aligning with global trends of earlier PCOS onset driven by childhood obesity, sedentary behavior, and metabolic decline [11,23]. Urbanization and Westernized diets exacerbate insulin resistance and hyperandrogenism [24]. Improved clinical awareness and diagnostic criteria also contribute to higher adolescent detection [7], highlighting adolescence as a critical intervention window for early screening and weight management [25].

Among 15–19-year-olds, incidence and prevalence increased 2.2-fold and 1.1-fold respectively. Adolescence is a pivotal window for PCOS symptom emergence, as hormonal and metabolic imbalances become evident [7,26]. This age group represents a key target for adolescent-specific interventions, including screening for menstrual irregularities, metabolic risk factors, and mental health symptoms [27]. Educational campaigns to improve awareness of PCOS-associated health risks, such as infertility, type 2 diabetes, and psychological disorders, are essential to prevent delays in diagnosis and care [8].

Conversely, women aged 20–29 years showed declining incidence but rising prevalence. This paradox may reflect successful early management reducing later symptomatic presentations. However, sustained prevalence growth underscores PCOS’s chronic nature, requiring lifelong comorbidity management [28].

While incidence and prevalence in older age groups (50–54 years) remained lower in absolute terms, a steady increase was observed over the study period. Incidence rose from 117 cases in 1990–307 cases in 2021, and prevalence increased from 83,652–379,602 cases. This trend may reflect improved survival rates, enhanced diagnosis, and recognition of PCOS-related complications. Although PCOS symptoms often decline after menopause, its metabolic and cardiovascular consequences persist [29], contributing to an elevated burden of type 2 diabetes, cardiovascular disease, and psychological disorders [28]. These findings highlight the importance of longitudinal care models that span the reproductive and post-reproductive life course [30].

The trends in DALYs mirror those observed for incidence and prevalence, with significant increases in younger age groups. DALYs for individuals aged 10–14 years rose from 1,079 in 1990–1,887 in 2021, reflecting the detrimental impact of early-onset PCOS on quality of life and metabolic health. A similar rise was observed in the 15–19 years group, underscoring the cumulative burden of untreated or poorly managed PCOS. By contrast, DALYs among older women remained comparatively low but increased steadily over time, reflecting the long-term consequences of PCOS-related comorbidities. These findings are consistent with global evidence indicating that women with PCOS face significantly higher rates of depression, anxiety, and cardiometabolic disorders [30], which together reduce quality of life and increase healthcare utilization [31,32].

Our decomposition analysis revealed that epidemiological changes were the primary driver of the rising burden of PCOS, accounting for 267.36% of the increase in incidence, 96.1% of the rise in prevalence, and 97.81% of the growth in DALYs. These changes reflect the growing influence of modifiable risk factors such as obesity, physical inactivity, and unhealthy diets, all of which are exacerbated by rapid urbanization [33]. Additionally, enhanced clinical awareness and improved diagnostic practices have contributed to increased detection rates, particularly among adolescents and young adults. Population growth also contributed to the rising burden, adding 91.51% to incidence, 30.68% to prevalence, and 30.83% to DALYs. While demographic expansion has increased the absolute number of women at risk for PCOS, our findings emphasize that the primary driver remains worsening disease risk at the individual level. In contrast, population aging exerted a mitigating effect, reducing incidence, prevalence, and DALYs by –258.87%, –26.78%, and –28.64%, respectively. This reflects the age-specific nature of PCOS, which primarily affects women of reproductive age [29]. However, as the population continues to age, there remains an unmet need to address the long-term metabolic and cardiovascular impacts of PCOS in post-reproductive women.

Projections indicate that the burden of PCOS will continue to rise substantially through 2035. The ASIR is expected to increase from 43.05 per 100,000 in 2021 to 70.82 per 100,000 by 2035, driven by worsening lifestyle risk factors, including obesity and sedentary behavior. Similarly, the ASPR is projected to rise from 1,165.03 per 100,000 in 2021–1,661.80 per 100,000, reflecting the chronic nature of PCOS and increasing exposure to metabolic risks in younger cohorts. Particularly concerning is the predicted acceleration of prevalence in women aged 10–24 years, highlighting the need for urgent interventions to address modifiable risk factors in adolescence [23]. The projected increase in DALYs, rising from 10.07 per 100,000 in 2021 to 14.35 per 100,000 by 2035, underscores the cumulative impact of PCOS on long-term health outcomes [11]. The interplay between PCOS, obesity, type 2 diabetes, and cardiovascular disease is expected to exacerbate the overall disease burden, particularly in older age groups [34,35]. Additionally, the significant mental health burden associated with PCOS requires greater attention in public health strategies to ensure comprehensive care. As GBD 2021 provides data only through 2021, projections beyond this year are based on statistical modeling rather than observed data. At the time of writing, there are no nationally representative population-based studies of PCOS burden in China for 2022–2025. Future studies validating these projections against real-world data would be valuable.

The escalating burden of PCOS necessitates a multifaceted, life-course approach to prevention, diagnosis, and management. Preventive strategies targeting obesity and sedentary behavior in childhood and adolescence are critical to reducing the early onset of PCOS. School-based interventions promoting healthy nutrition, physical activity, and early screening for metabolic risk factors should be prioritized [27]. For women of reproductive age, improved clinical awareness, early diagnosis, and timely interventions are essential to preventing long-term complications [8]. Access to diagnostic tools and healthcare services, particularly in underserved regions, must be expanded to ensure equitable care [36]. Comprehensive, integrated care models are required to address the reproductive, metabolic, and psychological dimensions of PCOS. Regular screening for type 2 diabetes, cardiovascular disease, and mental health conditions should be incorporated into primary care settings, particularly for women transitioning into post-reproductive years [37]. Addressing PCOS within existing non-communicable disease frameworks, alongside obesity and diabetes prevention programs, can enhance the efficiency and effectiveness of healthcare interventions.

### Strengths and limitations

This study has several notable strengths. First, we used data from the comprehensive GBD Study 2021, which employs consistent methods, enabling reliable trend analysis over 31 years. Second, our analysis includes projections to 2035, informing policymakers and healthcare providers for targeted interventions and resource allocation. Third, decomposition analysis disentangled the contributions of epidemiological changes, population growth, and aging, offering nuanced insights into PCOS burden drivers in China. This highlights modifiable lifestyle and environmental factors.

However, limitations exist. The GBD estimates rely on data availability and quality, which may vary across regions and time periods, potentially introducing uncertainty into our findings. Differences in diagnostic criteria and reporting practices for PCOS, particularly in earlier years, could have influenced incidence and prevalence estimates. Changes in diagnostic criteria over time, particularly the shift from NIH to Rotterdam criteria, may have contributed to increased detection rates. While GBD adjusts for such variations using meta-regression, residual bias may persist, potentially inflating temporal trends. Additionally, while the GBD framework adjusts for missing data and variability across studies, there remains the possibility of underestimating or overestimating disease burden, particularly in underserved or rural areas with limited healthcare access. Lastly, the inability to account for subtype variations in PCOS, such as phenotypic differences, which may affect the clinical and epidemiological understanding of the condition. Moreover, PCOS comprises multiple phenotypes-such as hyperandrogenic, ovulatory, and non-hyperandrogenic subtypes-that differ in clinical presentation, cardiometabolic risk, and long-term health outcomes. The inability of the GBD database to stratify PCOS burden by phenotype may obscure important differences in prevalence patterns and comorbidity risks. As hyperandrogenic phenotypes are often associated with more severe metabolic dysfunction and mental health burden, future studies distinguishing between subtypes will be crucial to better inform precision public health interventions.

## Conclusion

The rising burden of PCOS in China, driven by worsening lifestyle risk factors and demographic changes, presents a significant public health challenge. Projections through 2035 highlight the urgent need for targeted, age-specific interventions that address the root causes of PCOS, promote early diagnosis, and provide comprehensive, long-term care. A coordinated, evidence-based strategy is essential to mitigate the future burden of PCOS and improve health outcomes for affected women across the life course.

## References

[1] Lizneva D, Suturina L, Walker W, Brakta S, Gavrilova-Jordan L, Azziz R. Criteria, prevalence, and phenotypes of polycystic ovary syndrome. Fertil Steril. 2016;106(1):6–15. doi: 10.1016/j.fertnstert.2016.05.003 27233760

[2] Bozdag G, Mumusoglu S, Zengin D, Karabulut E, Yildiz BO. The prevalence and phenotypic features of polycystic ovary syndrome: a systematic review and meta-analysis. Hum Reprod. 2016;31:2841–55. 27664216 10.1093/humrep/dew218

[3] Yong E-L, Teoh WS, Huang ZW. Polycystic ovary syndrome v.2023: Simplified diagnostic criteria for an East Asian phenotype. Ann Acad Med Singap. 2023;52(12):669–78. doi: 10.47102/annals-acadmedsg.202369 38920160

[4] Khanna P, Kumaresan M. Comprehensive Care for Women with Diabetes Mellitus and Gynecological Complications. In: Management of Diabetic Complications. Singapore: Springer Nature Singapore; 2024. p. 193–204.

[5] Choudhury AA, Devi Rajeswari V. Gestational diabetes mellitus - A metabolic and reproductive disorder. Biomed Pharmacother. 2021;143:112183. 34560536 10.1016/j.biopha.2021.112183

[6] Greenwood EA, Pasch LA, Shinkai K, Cedars MI, Huddleston HG. Clinical course of depression symptoms and predictors of enduring depression risk in women with polycystic ovary syndrome: Results of a longitudinal study. Fertil Steril. 2019;111:147–56. 30458991 10.1016/j.fertnstert.2018.10.004

[7] Peña AS, Witchel SF, Hoeger KM, Oberfield SE, Vogiatzi MG, Misso M, et al. Adolescent polycystic ovary syndrome according to the international evidence-based guideline. BMC Med. 2020;18(1):72. doi: 10.1186/s12916-020-01516-x 32204714 PMC7092491

[8] Fernandez RC, Moore VM, Rumbold AR, Whitrow MJ, Avery JC, Davies MJ. Diagnosis delayed: health profile differences between women with undiagnosed polycystic ovary syndrome and those with a clinical diagnosis by age 35 years. Hum Reprod. 2021;36(8):2275–84. doi: 10.1093/humrep/deab101 33963388 PMC8289294

[9] Dadachanji R, Shaikh N, Mukherjee S. Genetic Variants Associated with Hyperandrogenemia in PCOS Pathophysiology. Genet Res Int. 2018;2018:7624932. doi: 10.1155/2018/7624932 29670770 PMC5835258

[10] Yu J, Zhou Y, Ding J, Zhang D, Yu C, Huang H. Characteristics and possible mechanisms of metabolic disorder in overweight women with polycystic ovary syndrome. Front Endocrinol (Lausanne). 2023;13:970733. doi: 10.3389/fendo.2022.970733 36714563 PMC9878688

[11] Zhang J, Zhu Y, Wang J, Hu H, Jin Y, Mao X, et al. Global burden and epidemiological prediction of polycystic ovary syndrome from 1990 to 2019: A systematic analysis from the Global Burden of Disease Study 2019. PLoS One. 2024;19(7):e0306991. doi: 10.1371/journal.pone.0306991 39024211 PMC11257291

[12] GBD 2021 Anaemia Collaborators. Prevalence, years lived with disability, and trends in anaemia burden by severity and cause, 1990-2021: findings from the Global Burden of Disease Study 2021. Lancet Haematol. 2023;10(9):e713–34. doi: 10.1016/S2352-3026(23)00160-6 37536353 PMC10465717

[13] Meng Y, et al. Global burden of polycystic ovary syndrome among women of childbearing age, 1990–2021: a systematic analysis using the global burden of disease study 2021. Front Public Health. 2025;13. 10.3389/fpubh.2025.1514250 PMC11979288 40206176

[14] GBD 2019 Respiratory Tract Cancers Collaborators. Global, regional, and national burden of respiratory tract cancers and associated risk factors from 1990 to 2019: a systematic analysis for the Global Burden of Disease Study 2019. Lancet Respir Med. 2021;9(9):1030–49. doi: 10.1016/S2213-2600(21)00164-8 34411511 PMC8410610

[15] Zheng, P., Barber, R., Sorensen, R., Murray, C. & Aravkin, A. Trimmed Constrained Mixed Effects Models: Formulations and Algorithms. 2020. Available from: 10.1101/2020.01.28.923599

[16] Vollset SE, Goren E, Yuan C-W, Cao J, Smith AE, Hsiao T, et al. Fertility, mortality, migration, and population scenarios for 195 countries and territories from 2017 to 2100: a forecasting analysis for the Global Burden of Disease Study. Lancet. 2020;396(10258):1285–306. doi: 10.1016/S0140-6736(20)30677-2 32679112 PMC7561721

[17] Kim HJ, Fay MP, Feuer EJ, Midthune DN. Permutation tests for joinpoint regression with applications to cancer rates. Stat Med. 2000;19(3):335–51. doi: 10.1002/(sici)1097-0258(20000215)19:3<335::aid-sim336>3.0.co;2-z 10649300

[18] Tay CT, et al. International evidence‐based polycystic ovary syndrome guideline update: insights from a systematic review and meta‐analysis on elevated clinical cardiovascular disease in polycystic ovary syndrome. JAHA. 2024;13. 10.1161/JAHA.123.033572 PMC11963914 39119982

[19] Yang G, Hu J, Rao KQ, Ma J, Rao C, Lopez AD. Mortality registration and surveillance in China: History, current situation and challenges. Popul Health Metr. 2005;3(1):3. doi: 10.1186/1478-7954-3-3 15769298 PMC555951

[20] Yang Y, Schulhofer‐Wohl S, Fu WJ, Land KC. The Intrinsic Estimator for Age‐Period‐Cohort Analysis: What It Is and How to Use It. Am J Sociol. 2008;113(6):1697–736. doi: 10.1086/587154

[21] Gelman A, Rubin DB. Inference from iterative simulation using multiple sequences. Statist Sci. 1992;7.

[22] Riebler A, Held L. Projecting the future burden of cancer: Bayesian age-period-cohort analysis with integrated nested Laplace approximations. Biom J. 2017;59(3):531–49. doi: 10.1002/bimj.201500263 28139001

[23] Patten R. Effect of exercise interventions on metabolic, reproductive and mental health in overweight women with polycystic ovary syndrome. 2021.

[24] Wang J, Wu D, Guo H, Li M. Hyperandrogenemia and insulin resistance: The chief culprit of polycystic ovary syndrome. Life Sci. 2019;236:116940. doi: 10.1016/j.lfs.2019.116940 31604107

[25] Zeng X, Xie Y-J, Liu Y-T, Long S-L, Mo Z-C. Polycystic ovarian syndrome: Correlation between hyperandrogenism, insulin resistance and obesity. Clin Chim Acta. 2020;502:214–21. doi: 10.1016/j.cca.2019.11.003 31733195

[26] Sultan C, Gaspari L, Hamamah S, Paris F. Adolescence: A High-Risk Period for PCOS Development? In: ISGE Series 13-24. Cham: Springer International Publishing; 2021. p. 13–24. doi: 10.1007/978-3-030-63650-0_2

[27] Conlon JL. Diagnosis and Management of Adolescents with Polycystic Ovary Syndrome in Primary Care: A Mixed Method Study to Explore Provider Behaviors and Barriers and Facilitators to Practice. George Washington University; 2021.

[28] Zore T, Joshi NV, Lizneva D, Azziz R. Polycystic Ovarian Syndrome: Long-Term Health Consequences. Semin Reprod Med. 2017;35(3):271–81. doi: 10.1055/s-0037-1603096 28658711

[29] de Medeiros SF, Yamamoto MMW, Souto de Medeiros MA, Barbosa BB, Soares JM, Baracat EC. Changes in clinical and biochemical characteristics of polycystic ovary syndrome with advancing age. Endocr Connect. 2020;9(2):74–89. doi: 10.1530/EC-19-0496 31905164 PMC6993261

[30] Sanchez N. A life course perspective on polycystic ovary syndrome. Int J Womens Health. 2014;6:115–22. doi: 10.2147/IJWH.S55748 24489477 PMC3904822

[31] Dokras A, Stener-Victorin E, Yildiz BO, Li R, Ottey S, Shah D, et al. Androgen Excess- Polycystic Ovary Syndrome Society: position statement on depression, anxiety, quality of life, and eating disorders in polycystic ovary syndrome. Fertil Steril. 2018;109(5):888–99. doi: 10.1016/j.fertnstert.2018.01.038 29778388

[32] Karjula S. Long-term consequences of polycystic ovary syndrome on mental health and health-related quality of life. University of Oulu; 2021.

[33] Awoke MA. Weight gain and lifestyle factors in women with and without polycystic ovary syndrome. Hum Reproduct. 2021;37:129–41. 10.1093/humrep/deab239 34788426

[34] Orio F, et al. Obesity, type 2 diabetes mellitus and cardiovascular disease risk: an uptodate in the management of polycystic ovary syndrome. Europ J Obstetr Gynecol Reproduct Biol. 2016;207:214–9. 10.1016/j.ejogrb.2016.08.026 27575870

[35] Zhu T, Cui J, Goodarzi MO. Polycystic Ovary Syndrome and Risk of Type 2 Diabetes, Coronary Heart Disease, and Stroke. Diabetes. 2021;70(2):627–37. doi: 10.2337/db20-0800 33158931

[36] Alenzi EO, Alqntash NH, Almajed EH, AlZabin AK. Risk of polycystic ovary syndrome: a population-based analysis of sociodemographic factors, healthcare access, health behaviors, and health status. BMC Womens Health. 2024;24(1):623. doi: 10.1186/s12905-024-03446-9 39580400 PMC11585150

[37] Moran LJ, Misso ML, Wild RA, Norman RJ. Impaired glucose tolerance, type 2 diabetes and metabolic syndrome in polycystic ovary syndrome: a systematic review and meta-analysis. Hum Reprod Update. 2010;16(4):347–63. doi: 10.1093/humupd/dmq001 20159883

